# Current status and future prospects of molecular imaging in targeting the tumor immune microenvironment

**DOI:** 10.3389/fimmu.2025.1518555

**Published:** 2025-01-22

**Authors:** Xiang Wang, Weifen Shen, Lingjun Yao, Chao Li, Huiming You, Duancheng Guo

**Affiliations:** ^1^ Department of Radiology, First People’s Hospital of Linping District, Hangzhou, China; ^2^ The Second Affiliated Hospital of Zhejiang University School of Medicine, Hangzhou, China; ^3^ Cancer Institute, Fudan University Shanghai Cancer Center, Shanghai, China; ^4^ Department of Oncology, Shanghai Medical College, Fudan University, Shanghai, China

**Keywords:** molecular imaging, tumor microenvironment, tumor immune, immunotherapy, immune cells

## Abstract

Molecular imaging technologies have significantly transformed cancer research and clinical practice, offering valuable tools for visualizing and understanding the complex tumor immune microenvironment. These technologies allow for the non-invasive examination of key components within the tumor immune microenvironment, including immune cells, cytokines, and stromal cells, providing crucial insights into tumor biology and treatment responses. This paper reviews the latest advancements in molecular imaging, with a focus on its applications in assessing interactions within the tumor immune microenvironment. Additionally, the challenges faced by molecular imaging technologies are discussed, such as the need for highly sensitive and specific imaging agents, issues with data integration, and difficulties in clinical translation. The future outlook emphasizes the potential of molecular imaging to enhance personalized cancer treatment through the integration of artificial intelligence and the development of novel imaging probes. Addressing these challenges is essential to fully realizing the potential of molecular imaging in improving cancer diagnosis, treatment, and patient outcomes.

## 1 Introduction

Tumor immunotherapy has transformed the way cancer is treated by utilizing the immune system’s capacity to identify and destroy cancerous cells. Immunotherapy functions by enhancing or reinstating the immune system’s capacity to combat cancer, in contrast to conventional treatments that specifically target cancer cells (1). This strategy encompasses multiple approaches, including cancer vaccines, adoptive cell transfer, and immune checkpoint inhibitors, each of which offers a distinct mechanism to improve anti-tumor immunity (2, 3). By preventing the inhibitory mechanisms that cancer cells employ to avoid immune detection, immune checkpoint inhibitors, such as PD-1/PD-L1 and CTLA-4 inhibitors, have shown extraordinary success in treating a variety of malignancies (4, 5). Patients with cancers that were previously incurable now have far higher survival rates thanks to these treatments. Furthermore, the efficiency of immunotherapy has been further increased by developments in biomaterials, such as nano-delivery systems, which improve the targeted distribution of therapeutic drugs, lowering systemic toxicity and enhancing patient outcomes (6). By combining these cutting-edge techniques, cancer treatment is being stretched to new heights, with the prospect of better patient outcomes and sustained remission. Tumor immunotherapy has the potential to revolutionize cancer treatment by offering new, less hazardous, and more efficient treatment choices as research into it advances.

Molecular imaging has the potential to revolutionize medical diagnosis and research by providing a means of seeing, characterizing, and quantifying biological processes occurring at the molecular and cellular levels in live creatures (7). In contrast to conventional imaging methods, molecular imaging offers valuable insights into the fundamental mechanisms of diseases, hence promoting timely identification, accurate diagnosis, and the assessment of therapeutic effectiveness. The creation of innovative molecular imaging methods and probes is one of the major developments in this area (8). Comparably, immune cell dynamics in cancer patients have been visualized by PET imaging with new radiotracers, which has aided in the noninvasive evaluation of treatment responses (9). Furthermore, the use of ^124^I-labeled antibodies has demonstrated potential in focusing on particular immune cells for PET imaging, allowing for a thorough view of activated T cells *in vivo* (10). These developments highlight the critical role molecular imaging plays in expanding our knowledge of disease mechanisms, increasing the precision of diagnostics, and developing individualized treatment plans.

Targeted tumor immunotherapy is advancing due to the incorporation of new molecular imaging agents and methodologies. These developments improve the accuracy and efficacy of therapeutic interventions and deepen our understanding of tumor-immune interactions. With enhanced monitoring of treatment responses, more precise diagnoses, and the creation of customized immunotherapy plans, molecular imaging technologies have the potential to revolutionize cancer treatment. This article will review the development of molecular imaging techniques in recent years, the role of molecular imaging in the tumor immune microenvironment, and its application in tumor immunotherapy. Additionally, it explores the challenges and future development trends of molecular imaging technology.

## 2 Development of molecular imaging technologies

### 2.1 Optical imaging

Optical imaging has become an essential tool in molecular imaging, providing a fine-grained understanding of biological processes at the molecular and cellular levels. This technology encompasses various techniques, including optical coherence tomography, bioluminescence imaging, and fluorescence imaging, each offering unique advantages for research and therapeutic applications. Fluorescence and bioluminescence imaging have garnered significant attention due to their increased sensitivity, non-radiative nature, and cost-effectiveness compared to traditional imaging modalities. These methods enable real-time monitoring of biological processes in living organisms, making them indispensable for tumor research and drug development (11). For instance, fluorescence molecular tomography (FMT) and bioluminescence tomography (BLT) facilitate three-dimensional imaging of molecular and cellular processes *in vivo*, enhancing our understanding of tumor biology and treatment efficacy (12). The diagnostic potential of optical imaging is enhanced when combined with other imaging modalities, such as nuclear medicine. Hybrid systems that integrate optical and nuclear imaging techniques, such as photonic-based nuclear medicine detectors and Cerenkov luminescence imaging, provide comprehensive insights into the molecular foundations of diseases (13). The resolution and sensitivity of optical imaging systems have also improved in recent years. High-resolution imaging techniques, such as optical coherence tomography and reflectance confocal microscopy, have significantly enhanced our ability to identify and diagnose diseases at the cellular and molecular levels (14).

The characterization and monitoring of various molecular events *in vivo* have been facilitated by the development of novel fluorescent protein-based genetically encoded probes and lipoprotein-based nanocarriers, aiding in the early diagnosis of tumors and the development of new drugs (15). Tansi et al. highlights the use of bispecific liposomes that target the tumor microenvironment, enabling effective tumor imaging and therapeutic applications. These liposomes, which target both fibroblast activation protein (FAP) and endoglin, facilitate enhanced tumor detection and selective drug delivery (16). Similarly, Li et al. developed engineered near-infrared fluorescent protein assemblies that improve both *in vivo* imaging and drug delivery in cancer treatment (17). Optical imaging enhances molecular imaging by matching the sensitivity performance of nuclear medicine techniques, particularly in visualizing biomarker expression levels (18). Additionally, near-infrared (NIR) optical imaging enables the noninvasive assessment of significant molecular events *in vivo*, including the imaging of cell surface receptors, proteases, and apoptosis in conditions such as rheumatoid arthritis, cancer, and cardiovascular disease (19). Optical imaging is a highly effective modality in molecular imaging due to its versatility, sensitivity, and high resolution. It continues to push the boundaries of clinical diagnostics and biomedical research, providing in-depth, up-to-date understanding of the intricate molecular mechanisms underpinning both health and disease.

### 2.2 MRI

Magnetic Resonance Imaging (MRI) is a non-invasive imaging technique that utilizes strong magnetic fields and radiofrequency waves to produce detailed images of internal body structures. It excels in differentiating various types of soft tissues, making it particularly valuable in neurological, musculoskeletal, cardiovascular, and oncological imaging. MRI operates by exploiting the magnetic properties of hydrogen atoms, which are abundant in the human body. When placed in a strong magnetic field, the nuclei of hydrogen atoms align with the field (19). Radiofrequency pulses are then used to disturb this alignment, and as the nuclei return to their original state, they emit signals that are detected and used to construct images. The resulting images have high spatial resolution and excellent contrast between different types of soft tissues. One of the significant advancements in MRI is the development of advanced imaging modalities such as multi-parametric MRI sequences, diffusion tensor imaging (DTI), and functional MRI (fMRI). These techniques allow for comprehensive characterization of complex conditions like glioblastomas, providing critical insights into tumor microenvironments and aiding in personalized therapy approaches (20). Additionally, fMRI and tractography are increasingly used to map brain functions and critical pathways, minimizing postsurgical neuro-deficits. MRI perfusion imaging has emerged as a powerful tool in assessing tumor microvascular structure and physiology. Techniques such as dynamic susceptibility contrast (DSC) and dynamic contrast enhancement (DCE) MRI provide quantitative imaging of tumor perfusion, which is crucial for treatment planning and monitoring (21). Hybrid imaging systems that combine MRI with PET or CT offer comprehensive diagnostic capabilities by providing both anatomical and functional information. This integration is particularly beneficial in oncology, where detailed imaging of tumor metabolism and structure is required for accurate diagnosis and treatment planning (22).Due to its unique imaging capabilities, MRI is extensively used in diagnosing and managing neurological, cardiovascular, and musculoskeletal diseases, as well as in oncology. It provides critical insights into the complex biological processes underlying various diseases.

### 2.3 SPECT

Single Photon Emission Computed Tomography (SPECT) is an advanced nuclear imaging technology that provides three-dimensional images of functional processes within the body. By utilizing gamma-emitting radioisotopes and gamma cameras, SPECT offers valuable insights into various physiological and pathological conditions (23). When gamma rays are emitted from within the patient, they are detected by gamma cameras rotating around the patient. The collected data are then reconstructed into three-dimensional images, providing detailed information about the distribution and concentration of the radiopharmaceutical within the body. This enables high-specificity visualization of functional processes such as blood flow, metabolism, and receptor binding. SPECT/CT imaging combines the functional information provided by SPECT with the anatomical details from CT, resulting in more accurate localization and characterization of lesions (24). This hybrid imaging modality is particularly valuable in tumor imaging, lymphoma staging, and dosimetry calculations for radiotherapy (25). Additionally, SPECT has found applications in cardiovascular imaging, infection and inflammation imaging, and cerebral vasculitis. The combination of SPECT with CT, along with the development of new imaging techniques and radiopharmaceuticals, has enhanced its accuracy and utility across various medical fields, including oncology, neurology, and cardiology.

### 2.4 PET

Positron Emission Tomography (PET) operates by detecting gamma rays indirectly emitted by positron-emitting radiotracers, the most commonly used being fluorodeoxyglucose (FDG), a glucose analog. When the radiotracer decays, it emits positrons that collide with electrons, resulting in the emission of gamma photons. These photons are detected by the PET scanner, which reconstructs the data into three-dimensional images, providing detailed information about metabolic activity and function. PET imaging plays a crucial role in monitoring responses to immunotherapy in various cancers, including lung adenocarcinoma, Hodgkin’s lymphoma, and breast cancer. It offers non-invasive whole-body imaging, aiding in the assessment of treatment response and tumor heterogeneity (26–28). Recent studies highlight the use of PET radiomics to visualize tumor-infiltrating CD8+ T cell exhaustion, optimizing radiotherapy and immunotherapy combinations in lung cancer models (29). This approach enhances understanding of tumor immune dynamics, aiding in the development of personalized treatment strategies. The latest developments in CD8-PET/CT imaging provide a non-invasive alternative to assess CD8+ tumor infiltration levels in patients undergoing immunotherapy for solid tumors, offering crucial insights for predicting immunotherapy response (30). Additionally, studies have found that Total Metabolic Tumor Volume (TMTV) on 18F-FDG PET/CT serves as an important prognostic biomarker in patients with extensive small cell lung cancer undergoing first-line chemo-immunotherapy, with strong predictive value for overall survival and progression-free survival (31). The development of immuno-PET imaging using tumor-specific Major Histocompatibility Complex II (tsMHC-II) to predict immunotherapy response, particularly checkpoint inhibitor therapy in melanoma, represents a significant advancement. Immuno-PET can provide valuable insights into the dynamic changes in tsMHC-II expression related to treatment response, aiding in predicting therapeutic outcomes (32). The integration of PET with immuno-oncology offers new insights into tumor-immune interactions and enhances the precision of therapeutic interventions. As technology advances, PET will continue to be a key tool in medical imaging, providing critical insights into the functional processes of various diseases.

### 2.5 Ultrasound imaging

Ultrasound imaging uses high-frequency sound waves to generate images of internal structures. The transducer emits sound waves that pass through the body and reflect off tissues and organs. These echoes are captured and converted into real-time images. Ultrasound is non-invasive, does not use ionizing radiation, and provides detailed information about soft tissue structures. Recent studies suggest that measuring tumor stiffness and perfusion using ultrasound imaging can predict responses to cancer immunotherapy. This method shows a strong linear correlation between tumor stiffness (elastic modulus) and perfusion parameters (33). Ultrasound imaging is also used to temporarily disrupt the blood-brain barrier and blood-tumor barrier, improving the delivery and efficacy of brain tumor immunotherapy by temporarily increasing the permeability of these barriers (34). Researchers have used CD93-targeted microbubbles in ultrasound imaging to predict the tumor immune microenvironment, aiding immunotherapy decision-making. This method provides a non-invasive approach to assess and monitor the tumor immune status (35). Ultrasound imaging can also monitor vascular changes after tumor radiotherapy, which are associated with immune cell infiltration. This technique offers a non-invasive way to track the effectiveness of tumor immunotherapy (36). With advancing technology, ultrasound imaging methods are evolving. For instance, a new contrast agent has been developed for synergistic chemophotothermal therapy and enhanced immunotherapy of liver cancer and its metastases, showing significant efficacy in preclinical studies (37). Researchers have developed a multifunctional high-intensity focused ultrasound (HIFU)-specific metal-organic framework nano system that effectively inhibits tumor growth and eliminates lung metastases in preclinical models by combining HIFU with hypoxia-activated chemoimmunotherapy, representing a new cancer treatment strategy (38). Focused ultrasound (FUS) in both thermal ablation and mechanical forms can enhance tumor immunotherapy by improving immune cell infiltration and treatment penetration. This method has shown promising results in various preclinical and clinical trials (39). With continuous innovations in imaging technologies and contrast agents, ultrasound imaging will remain a key tool in medical imaging, providing crucial insights into the functional processes of various diseases.

### 2.6 Multimodal imaging

Multimodal imaging combines two or more imaging techniques to provide comprehensive information about tissue structure and function. Common combinations include CT, MRI, PET, ultrasound, and optical imaging. Each technology has its unique advantages; for example, MRI offers excellent soft tissue contrast, while PET provides functional and metabolic information. By integrating these technologies, multimodal imaging enhances diagnostic accuracy and provides detailed insights into disease processes.

**Figure 1:**
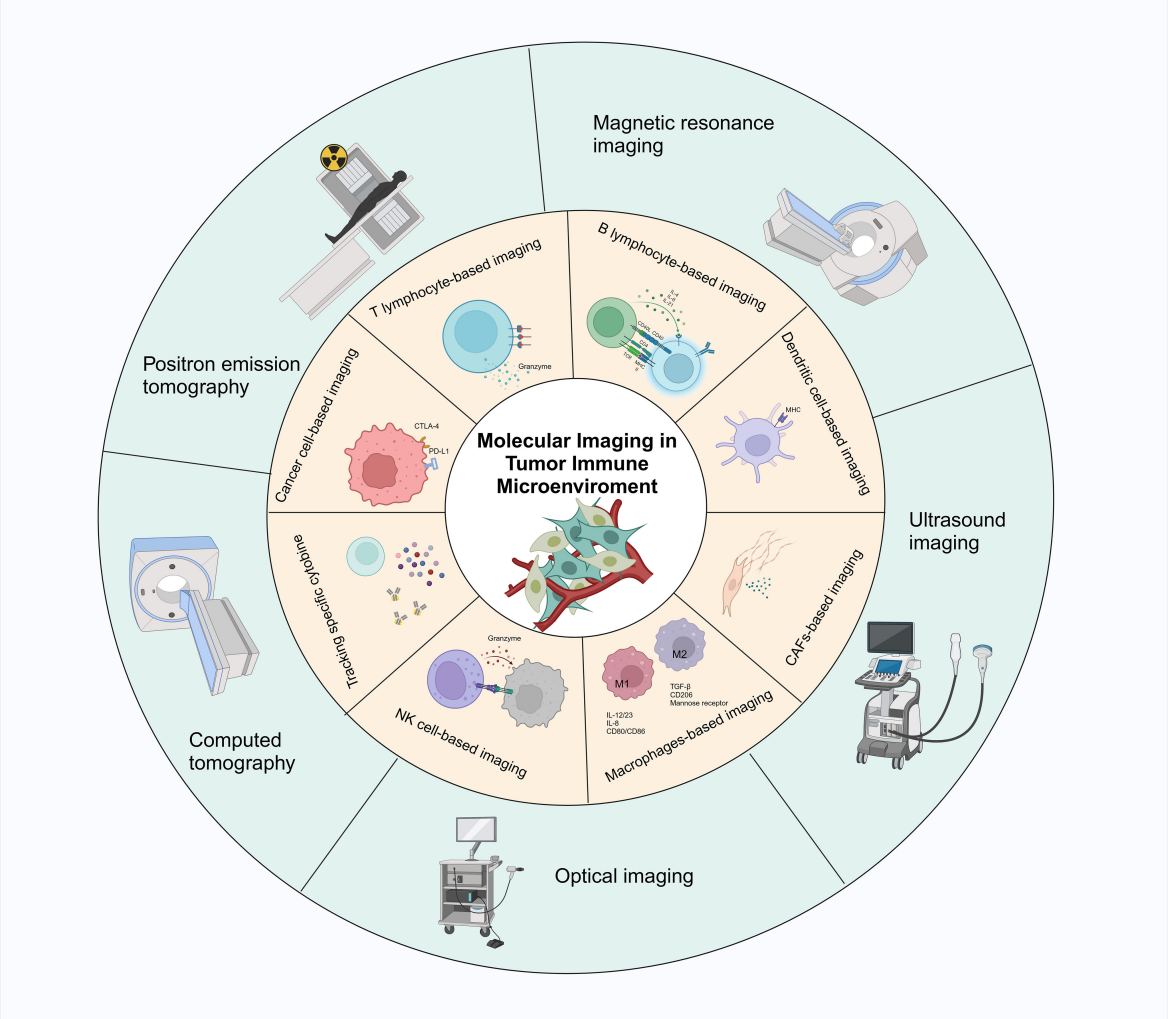
Schematic representation of different techniques for real-time monitoring of immune cells and immune signaling molecules in the immune microenvironment through molecular imaging. The main techniques include optical imaging, positron emission tomography (PET), magnetic resonance imaging (MRI), computed tomography (CT), and ultrasound imaging.

Recent research has developed integrated multimodal imaging and synergistic therapeutic nanoplatforms for precise tumor nanomedicine (40, 41). These platforms enable enhanced tri-modal photothermal-chemotherapy-immunotherapy, demonstrating their potential in comprehensive tumor treatment and monitoring (42). Multifunctional nanoplatforms combining CAR-T cell immunotherapy and chemo-photothermal therapy have shown promise in multimodal imaging-guided tumor immunotherapy applications. These platforms enhance therapeutic efficacy by promoting lymphoid tissue angiogenesis and improving CAR-T cell infiltration in solid tumors (43). Additionally, studies indicate that the multifunctional theranostic agent TPA-BT-DPTQ can be used for multimodal imaging, including fluorescence imaging (FLI), photoacoustic imaging (PAI), and photothermal imaging (PTI). This agent combines photothermal therapy and immune effects, enhancing the efficacy of immunotherapy in inhibiting tumor growth and preventing cancer metastasis and recurrence (44). Advances in MRI imaging, combined with radiomics and deep learning, facilitate the assessment of tumor response to immunotherapy. This combination explores the biological characteristics of hepatocellular carcinoma (HCC), aiding in personalized treatment planning (40). The integration of various imaging technologies and the development of novel theranostic agents offer new insights into tumor-immune interactions and enhance the precision of therapeutic interventions (**Figure 1**).

## 3 Molecular imaging in targeting the tumor immune microenvironment

### 3.1 Immune cells

#### 3.1.1 T cells

T cells are a crucial component of the immune system and play a key role in tumor immunotherapy. Cytotoxic T lymphocytes (CTLs), also known as CD8+ T cells, are essential for directly killing cancer cells (45). They recognize tumor antigens presented by MHC class I molecules through their T cell receptors (TCRs). Once activated, CTLs release cytotoxic granules containing perforin and granzymes, which induce apoptosis in target cells. CTLs also produce cytokines such as IFN-γ and TNF-α, further enhancing the anti-tumor immune response (46). CD4+ T cells, or helper T cells, support anti-tumor immunity by coordinating immune responses. They assist in activating CTLs and B cells and regulate TME by secreting various cytokines. CD4+ T cells can differentiate into subsets, including Th1, Th2, Th17, and regulatory T cells (Tregs), each playing different roles in immune regulation. For example, Th1 cells promote CTL responses by producing IFN-γ, while Tregs maintain tolerance and prevent autoimmunity by suppressing immune responses through cytokines like IL-10 and TGF-β, and contact-dependent mechanisms (47, 48). Tregs are critical in maintaining immune homeostasis and preventing autoimmune reactions. They inhibit effective anti-tumor immune responses by suppressing CTLs and other effector immune cells (49, 50). Memory T cells provide long-lasting anti-tumor immunity by remembering previously encountered tumor antigens. Upon re-exposure to the same antigen, they rapidly mount a robust immune response, offering long-term protection against tumor recurrence (51). Natural killer T (NKT) cells exhibit properties of both T cells and natural killer (NK) cells, recognizing lipid antigens presented by CD1d molecules. They participate in tumor immunity by producing cytokines that activate other immune cells and directly killing tumor cells (52, 53).

Molecular imaging technologies have revolutionized the monitoring and evaluation of tumor immunotherapy. These non-invasive imaging methods allow visualization of biological processes at molecular and cellular levels, aiding in treatment assessment and optimization. PET imaging combined with radiolabeled antibodies is widely used to track T cells *in vivo*. For instance, ^124^I-Basiliximab has been developed for CD25-targeted immuno-PET imaging, showing promising results in distinguishing activated from non-activated human peripheral blood mononuclear cells (10, 54). Additionally, a humanized radiolabeled CD8-targeted minibody has been used to quantify tumor-infiltrating CD8+ T cells in brain tumors via PET imaging (55). Another study used PET imaging to monitor CD4+ T cell recovery during combined immunotherapy and chemotherapy in triple-negative breast cancer (56). A novel SPECT/CT imaging probe, 99mTc-sum IL-2, targets IL-2Rβ/IL-2Rγ receptors on tumor-infiltrating T cells, showing potential in predicting immune responses to anti-PD-L1 therapy and tracking adoptively transferred (ACT) T cells (57). Optical imaging techniques, including fluorescence and bioluminescence imaging, are used to study T cell behavior in real-time. Rare-earth nanoparticles have been developed for non-invasive *in vivo* imaging of tumor-infiltrating T cells, providing valuable information for early identification of non-responders and improved treatment outcomes (58). X-ray fluorescence imaging (XFI) has demonstrated potential in tracking T cells labeled with nanoparticles, offering a non-invasive method to monitor T cell distribution and activity within tumors, aiding in evaluating immunotherapy efficacy (59). Whole-body dynamic imaging and kinetic modeling of ^18^F-AraG have been used to study T cell distribution and activation in healthy individuals and cancer patients undergoing immunotherapy. This method provides detailed insights into immune cell activity and treatment response (60).

Chimeric Antigen Receptor (CAR) T cell therapy is a groundbreaking immunotherapeutic approach in which T cells are genetically engineered to express CARs that specifically recognize and target cancer cells (61). Recent studies have leveraged advanced imaging techniques to monitor the biodistribution and therapeutic effects of CAR-T cells. PET imaging using ^89^Zr-labeled CAR-T cells has facilitated real-time tracking of their distribution within tumors. For example, the study by Volpe et al. demonstrated the use of ^89^Zr-labeled CAR-T cells to assess their biodistribution and kinetics *in vivo*, revealing their accumulation in organs such as the liver and lungs shortly after infusion. This technique provides crucial insights into the effectiveness of CAR-T cell therapy and helps optimize treatment protocols (62).In addition, FDG-PET imaging has proven invaluable in assessing the response to CAR-T therapy, particularly in lymphoma patients. This imaging modality has been employed to monitor treatment efficacy and predict the onset of cytokine release syndrome (CRS) (63). These imaging techniques not only track the migration and persistence of CAR-T cells but also correlate with clinical outcomes, aiding in the management of therapeutic responses and potential toxicities.

Advances in molecular imaging significantly enhance our ability to visualize and understand T cell dynamics, offering new directions for optimizing cancer immunotherapy.

#### 3.1.2 B cells

B cells function as antigen-presenting cells (APCs) by capturing and processing antigens, which are then presented to T cells through major histocompatibility complex (MHC) class II molecules. This interaction is crucial for activating CD4+ helper T cells, which in turn help activate CD8+ cytotoxic T cells, thereby enhancing the overall anti-tumor immune response (64, 65). B cells also differentiate into plasma cells that produce antibodies specific to tumor antigens. These antibodies can directly neutralize tumor cells, mark them for destruction by other immune cells through antibody-dependent cellular cytotoxicity (ADCC), or activate the complement system to induce tumor cell lysis. Tumor-infiltrating B cells (TIL-Bs) have been shown to play significant roles in tumor control by promoting anti-tumor immunity and interacting with other immune cells within the tumor microenvironment (TME). TIL-Bs facilitate the formation of tertiary lymphoid structures (TLSs) within tumors, which are associated with better prognosis and improved responses to immunotherapy (66). However, in some cases, B cells can exhibit immunosuppressive functions that may promote tumor progression. Regulatory B cells (Bregs) suppress effective anti-tumor immune responses by producing immunosuppressive cytokines such as IL-10, inhibiting T cell activity, and promoting the expansion of regulatory T cells (Tregs) (67).

PET and SPECT are advanced imaging technologies used to visualize and track B cells *in vivo*. These techniques allow non-invasive monitoring of B cell distribution, activation, and function within the TME, providing insights into their role during immunotherapy. For example, CD69 PET imaging has been used to monitor immune activation induced by immunotherapy, detecting CD69 expression on B cells and other immune cells with high sensitivity (68).Near-infrared fluorescence imaging using specific antibodies conjugated with fluorescent dyes, such as Miltuximab^®^-IRDye800, shows promise in targeting and visualizing B cells in tumors. This technology demonstrated high probe accumulation in urothelial carcinoma, indicating its potential for molecular imaging of bladder cancer (69). High-throughput spatial molecular imaging enables detailed characterization of TLSs and lymphoid aggregates in various solid tumors at the single-cell and subcellular levels. This approach provides comprehensive insights into the spatial organization and functional state of B cells within the TME (70). B cells play diverse and crucial roles in tumor immunity, from antigen presentation and antibody production to regulating immune responses in the TME (71). Advances in molecular imaging technologies have significantly enhanced our ability to visualize and understand B cell dynamics.

#### 3.1.3 TAMs

Tumor-associated macrophages (TAMs) play dual roles in the tumor microenvironment (TME), promoting both tumor growth and suppression. The polarization state of TAMs determines their function: M1 macrophages exhibit anti-tumor activity, while M2 macrophages support tumor growth and immune evasion (72). TAMs enhance tumor invasion and metastasis by secreting growth factors, angiogenic factors, and matrix metalloproteinases, which promote tumor cell proliferation, angiogenesis, and extracellular matrix degradation. For instance, in colorectal cancer, the polarization state of TAMs significantly affects tumor growth and metastasis (72). TAMs create an immunosuppressive environment in the TME by secreting cytokines such as IL-10 and TGF-β and expressing immune checkpoint molecules like PD-L1, which inhibit the functions of cytotoxic T cells and natural killer (NK) cells (72). Exosome secretion by TAMs also plays a key role in suppressing anti-tumor immunity. Exosomes carrying high levels of PD-L1 can inhibit CD8+ T cell proliferation and function (73).

A significant advancement in tumor molecular imaging is the development of nanoparticle contrast agents targeting TAMs. One notable example is SDIO nanoparticles, which show great potential in specifically detecting M2-polarized TAMs using MRI. This advancement is promising for assessing the localization and function of TAMs in cancer diagnosis and therapy. By providing clearer images of TAM distribution and activity within tumors, SDIO nanoparticles may improve the accuracy and effectiveness of cancer imaging and subsequent treatment (74). Similarly, ^68^Ga-labeled mannose-functionalized serum albumin nanoparticles have been developed for non-invasive imaging of TAMs. These nanoparticles not only help visualize TAMs but also closely monitor immune cell enrichment in the TME and response to anti-PD1 therapy, making them valuable tools for diagnostic imaging and therapeutic monitoring (75). Another significant advancement is the use of peptide-based imaging probes for TAM imaging. The ^68^Ga-labeled M2pep peptide has garnered attention for its rapid and targeted accumulation in tumors, making it an effective non-invasive TAM imaging probe (76). In PET imaging, probes such as ^68^Ga-NOTA-COG1410 have made significant strides. This radioligand targets TREM2 on TAMs, providing a specific and effective PET probe for diagnosing gastrointestinal tumors, thereby enhancing the specificity and sensitivity of PET imaging for these cancers (77). Additionally, the RP832c peptide targets CD206 expressed on M2-like macrophages. This peptide has therapeutic properties that reprogram TAMs to an anti-tumor phenotype and serves as a PET imaging agent in mouse cancer models (78). MRI susceptibility imaging has emerged as a promising non-invasive method for quantifying and phenotyping TAMs, particularly in glioblastoma, offering a way to assess TAM presence and activity without invasive procedures (79). Moreover, mass spectrometry and multiplex imaging technologies provide new techniques for studying the TME, revealing differences in the presence and behavior of TAMs, vasculature, and tumor cells in various tumor regions (80). These advancements highlight the importance of imaging technologies in understanding and targeting TAMs in the TME, paving the way for improved diagnostic and therapeutic strategies.

#### 3.1.4 NK cells

Natural killer (NK) cells are a vital component of the innate immune system, known for their ability to recognize and destroy aberrant or infected cells without prior sensitization. This potent cytotoxic activity and capacity to regulate immune responses make NK cells promising targets for cancer immunotherapy (81). NK cells can directly kill tumor cells by releasing cytotoxic granules containing perforin and granzymes, inducing apoptosis in target cells (82). They also express death receptor ligands, such as TRAIL and FasL, which trigger apoptosis in tumor cells expressing the corresponding receptors (83). NK cells produce cytokines such as IFN-γ and TNF-α, which enhance the anti-tumor activity of other immune cells, including dendritic cells (DCs) and T cells. NK cells also interact with DCs to promote the maturation and activation of adaptive immune responses, thereby bridging innate and adaptive immunity (84). NK cells play a crucial role in antibody-dependent cellular cytotoxicity (ADCC), a process where antibodies bound to tumor cells facilitate their destruction by NK cells. This is mediated by the binding of the antibody’s Fc region to CD16 (FcγRIII) on NK cells, leading to the release of cytotoxic granules and cytokines (85). Advances in genetic engineering, such as CRISPR/Cas9 technology, have enhanced NK cell cytotoxicity and resistance to the immunosuppressive tumor microenvironment (86). NK cell therapies, including CAR-NK cells and NK cell engagers (NKCEs), are actively being developed and tested in clinical trials. These therapies aim to harness NK cells’ natural cytotoxicity and enhance their ability to target and destroy tumor cells (87). Combination therapies with immune checkpoint inhibitors, such as anti-PD-1, have shown promising results in preclinical models, highlighting the potential of NK cells in cancer immunotherapy.

CAR-NK cells have shown promising therapeutic potential in treating various cancers, including aggressive ovarian cancer. Recent studies have monitored HER2-targeted CAR-NK cells using humanized PET reporter gene imaging. This non-invasive method allows real-time tracking of CAR-NK cells within patients, providing important insights into their distribution and activity in the tumor microenvironment, which helps optimize CAR-NK cell therapies. NK-92 cells were genetically modified to express a HER2-targeted CAR, the bioluminescence imaging reporter Antares, and the sodium iodide symporter (NIS), enabling detailed imaging (88). Additionally, researchers have used ^89^Zr-oxine-labeled NK cells for PET imaging to track their migration and distribution for up to seven days post-infusion. Real-time tracking is crucial for understanding NK cell dynamics and their interactions with tumor cells, enhancing the effectiveness of NK cell therapies (89). The molecular imaging agent [^18^F] AlF-mNOTA-GZP can detect changes in immune cell populations expressing granzyme B (such as GZB+ CD8+ T cells and GZB+ NK cells), further deepening our understanding of immune responses and treatment efficacy (90). Pham et al. utilized PET imaging to investigate the migration of ^89^Zr-labeled NK cells to HER2-positive breast tumors and assess the effects of trastuzumab treatment. The ^89^Zr-NK cells preserved their functional properties and predominantly accumulated in the liver and spleen. Treatment with trastuzumab increased the presence of NK cells within the tumor, demonstrating the effectiveness of this method for tracking NK cell migration (91).

A significant advancement in NK cell imaging is using MRI to track labeled NK cells. Researchers have used superparamagnetic iron oxide nanoparticles to label NK cells, enabling their visualization *in vivo* and clearly demonstrating NK cell migration to tumor sites. This method allows real-time monitoring of NK cell distribution and accumulation (92). Another study demonstrated the non-invasive detection and quantification of intratumoral NK cells using ^19^F MRI. This technology allows precise tracking of NK cell persistence and viability, highlighting its potential for real-time monitoring of NK cell therapies (93). Recent research has found that biomimetic nanoparticles can be used to label NK cells, facilitating effective MRI tracking. This approach not only improves NK cell visualization but also enhances their therapeutic efficacy by ensuring targeted delivery to tumor sites (94). Advances in molecular imaging technologies have significantly improved our understanding of NK cell dynamics, distribution, and interactions within the tumor microenvironment.

#### 3.1.5 Dendritic cells

Dendritic cells (DCs) are a crucial component of the immune system, serving as potent antigen-presenting cells (APCs) that bridge innate and adaptive immunity. They play a key role in tumor immunity (95). DCs efficiently capture and process tumor antigens, presenting them to T cells via major histocompatibility complex (MHC) molecules. DC-mediated cross-priming is essential for generating anti-tumor CD8+ T cell immunity, which is the cornerstone of effective immunotherapy (96). Type 2 conventional dendritic cells (cDC2s) are particularly important in supporting cytotoxic T cell responses and helper T cell differentiation. They are involved in both anti-tumor and pro-tumor immune responses, making them critical targets for cancer vaccination and cDC2-targeted immunotherapy (97). DCs also play a significant role in immune regulation. Blocking immune checkpoints such as Tim-3 can enhance the anti-tumor immunity of the STING agonist ADU-S100 by regulating the release of CD4+ T cells from cDC2s (98). This highlights the potential of combining checkpoint inhibitors with DC-targeted therapies to enhance anti-tumor responses. As potent activators of T cells and efficient antigen-presenting cells, DCs play a central role in tumor immunity. Therapies targeting DCs, including vaccines and metabolic reprogramming, offer promising prospects for enhancing cancer immunotherapy effectiveness (99).

MRI has emerged as a powerful tool for tracking DC vaccines. This imaging modality allows for the non-invasive determination of the accuracy of therapeutic DC injections and their subsequent migration to lymph nodes. By providing real-time visualization, MRI tracking serves as an early biomarker, potentially predicting the efficacy of tumor vaccination before clinical outcomes become apparent. This method significantly improves our understanding of DC distribution and their role in initiating immune responses (100). Autologous dendritic cells have been labeled with clinical superparamagnetic iron oxide preparations or In-oxine and injected intranodally under ultrasound guidance in melanoma patients. In contrast to scintigraphy, MRI assesses the accuracy of dendritic cell delivery as well as cell migration patterns between and within nodes (101). The development of engineered DC vaccines enables non-invasive monitoring of their spatiotemporal fate using CT and NIRF imaging. This technology allows real-time visualization of vaccine accumulation in draining lymph nodes and subsequent potent T cell responses. Advances in this imaging technology facilitate personalized tumor imaging and enhance the efficacy of radioimmunotherapy (102, 103). Hypoxia imaging using ^18^F-FMISO PET has been employed to guide hypoxia-targeted treatments for unresponsive tumors. This technology improves responses to immune checkpoint therapy by leveraging immune cell populations, such as dendritic cells, within the tumor microenvironment. The ability to identify hypoxic and immunosuppressive regions within tumors aids in developing more effective treatment strategies (104). Combined bioluminescence imaging and I-124 PET provides insight into the biological behavior of dendritic cells in living organisms and can be a useful tool for optimizing DC-based immunotherapy regimens (105). The development of advanced imaging technologies has greatly enhanced our ability to track and understand the role of DCs in cancer immunotherapy.

#### 3.1.6 Neutrophils

Neutrophils are the most abundant granulocytes in the blood and a key component of the innate immune system. They play a crucial role in defending against infections and can exhibit both pro-tumor and anti-tumor functions in tumorigenesis (106). In the tumor microenvironment (TME), neutrophils can display either an anti-tumor (N1) or pro-tumor (N2) phenotype. N1 tumor-associated neutrophils (TANs) are generally considered anti-tumor, while N2 TANs promote tumor growth and metastasis (107). Neutrophils facilitate tumor progression by secreting factors that support angiogenesis, extracellular matrix remodeling, and cancer cell proliferation. They also release neutrophil extracellular traps (NETs) that capture circulating tumor cells, promoting their distant metastasis (108). Additionally, neutrophils can induce T cell anergy, characterized by a shift in lipid transport pathways, which inhibits T cell proliferation and activation (109). Neutrophils contribute to creating a tumor-supportive microenvironment. For example, sTLR9^+ neutrophils are heavily recruited to tumor inoculation sites, promoting a microenvironment that supports tumor growth (110). Moreover, immature neutrophils in the bone marrow naturally exhibit immunosuppressive and pro-tumor activities (111). Targeting neutrophils as a therapeutic strategy includes modulating the CXCLs-CXCR2 axis to regulate their recruitment and function in the TME (112). Neutrophils exhibit functional plasticity, significantly influencing tumor biology through interactions with other immune cells and the TME.

In imaging, researchers have developed an activatable polymer probe for fluorescence and photoacoustic imaging of tumor-associated neutrophils (TANs). This innovation provides a new tool for TAN imaging, facilitating cancer immunotherapy drug screening and enhancing our understanding of TANs’ roles in cancer immunotherapy (113). Additionally, a lipid-based molecular probe, TFML, combined with engineered neutrophils, has shown promise in targeted photoacoustic imaging of brain tumors. This approach offers potential for neutrophil-based nanotherapy and improved brain tumor imaging (114). Furthermore, researchers have used antibody-conjugated superparamagnetic iron oxide particles (SPIOs) in magnetic particle imaging (MPI) to non-invasively and sensitively track neutrophils. This method allows for radiation-free imaging of inflammatory cells, providing a safe and effective way to monitor neutrophil dynamics (115). Combining neutrophil targeting with magnetic targeting has also increased the accumulation of photothermal agents at tumor sites, observed through MR imaging to visualize tumor location. Philipp Spatz et al. developed and preliminarily characterized the first ^18^F-CXCR2 radiotracer for PET imaging targeting neutrophils. This tracer, designed based on a phthalimide template and obtained through indirect labeling, demonstrated specificity and diagnostic potential in neutrophil imaging (116).

#### 3.1.7 MDSCs

Myeloid-derived suppressor cells (MDSCs) are a heterogeneous group of immune cells derived from myeloid progenitors that play a crucial role in tumor-induced immunosuppression (117). They exhibit strong immunosuppressive activity and promote tumor cell proliferation and invasion by secreting growth factors and enzymes, both directly and indirectly (118). Accumulating in the tumor microenvironment, MDSCs inhibit the function of anti-tumor effector cells such as T cells and NK cells, thereby weakening the body’s anti-tumor immune response. By suppressing the immune system, MDSCs enable tumor cells to evade immune detection and attack (119).

**Figure 2:**
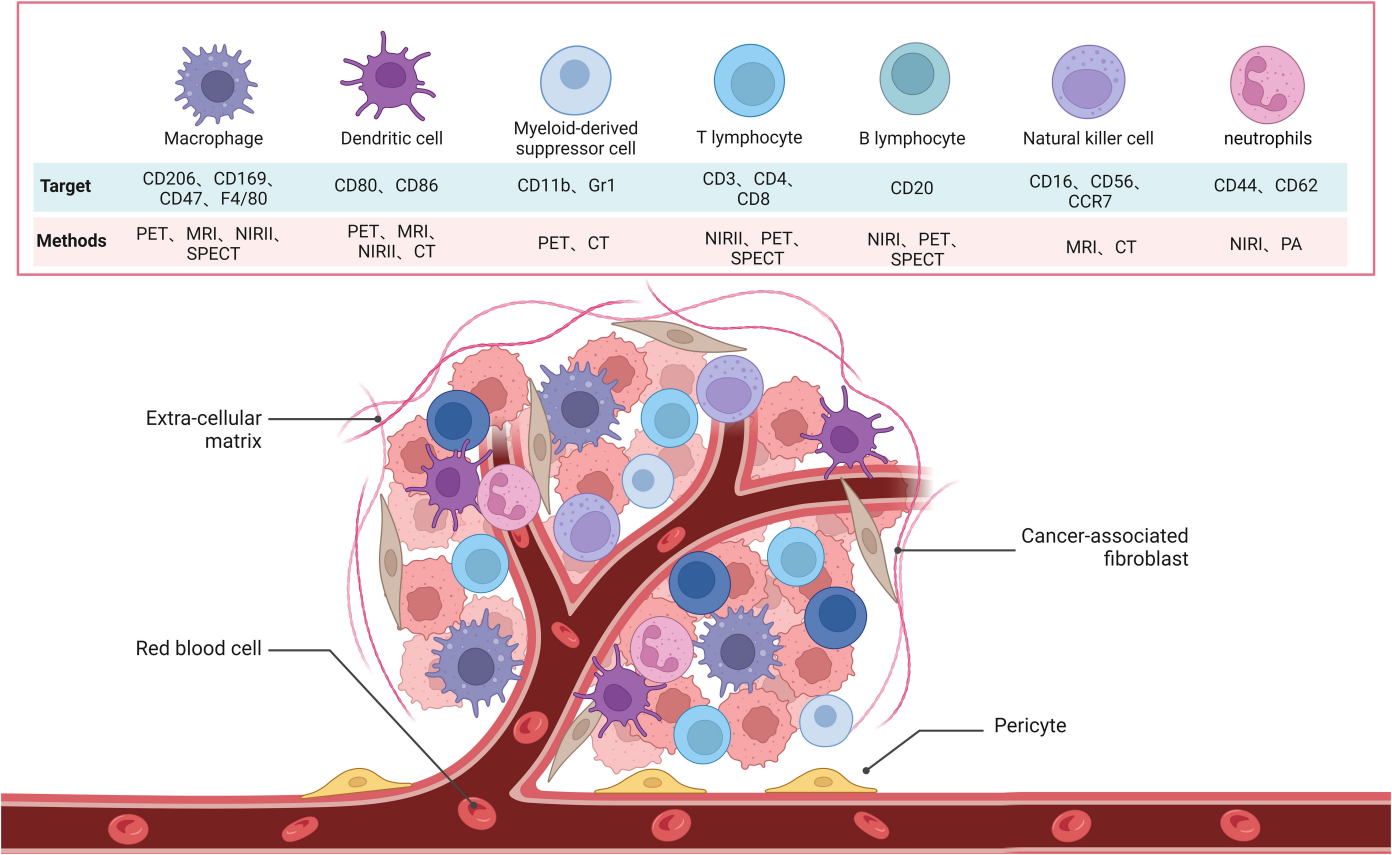
Schematic of the molecular images used to target immune cells, which include B cells, MDSC, NK cells, T cells, neutrophils, DCs and macrophages in TME.

PET imaging has been used to study the mobilization of monocytic MDSCs (M-MDSCs) induced by liver transplant injury, which promotes tumor recurrence through the CXCL10/TLR4/MMP14 signaling pathway. This imaging method offers potential therapeutic interventions to reduce post-liver transplant tumor recurrence (120). Additionally, studies have found a strong correlation between [^18^F] DPA-714 uptake and the quantity and activation level of glioma-associated myeloid cells (GAMs). TSPO expression is primarily limited to HLA-DR+ activated GAMs, particularly tumor-infiltrating HLA-DR+ MDSCs and TAMs. The findings suggest that [^18^F] DPA-714-PET can be used for non-invasive imaging of the immunosuppressive tumor microenvironment in gliomas and to characterize the heterogeneity of myeloid cell infiltration at different disease stages (121). Moreover, researchers have designed a biomimetic manganese-based theranostic nanoplatform for multimodal imaging and dual immunotherapy of cancer. This platform utilizes a manganese dioxide coating and MDSC cell membrane camouflage to target the tumor microenvironment for photothermal imaging, photoacoustic imaging, and magnetic resonance imaging, enhancing anti-tumor immunity through chemodynamic therapy and photothermal therapy (122). Advances in imaging technology have significantly improved our understanding of MDSCs and their role in tumor biology (**Figure 2**; **Table 1**).

**Table 1:** Imaging of immune cells.

Imaging modalities	Cell type	Tracer	Purpose	Ref
PET	T cell	^18^F-AraG	Study T cell distribution and activation in healthy individuals and cancer patients undergoing immunotherapy	(60)
PET	T cell	^124^I-Basiliximab	Promise results in distinguishing activated from non-activated human peripheral blood mononuclear cells	(10, 54)
PET	B cell	[^89^Zr]-DFO-H1.2F3	Detect CD69 expression on B cells	(68)
NIR	B cell	Miltuximab^®^-IRDye800	Promise in targeting and visualizing B cells in tumors	(69)
MRI	Macrophages	SPIO	Visualization of TAMs in breast cancer	(74)
PET	Macrophages	^68^Ga-labeled M2pep peptide	Focus on the rapid and targeted accumulation of macrophages in tumors	(76)
PET	NK cell	^18^F-FDG	Track CAR NK-92 cells localization to HER2/neu-positive tumors	(88)
MRI	NK cell	^19^F	Monitor NK cell migration in neuroblastoma and lymphoma xenografts	(93)
MRI	DC	SPIO	Track dendritic cells and response to DC vaccine in melanoma patients	(101)
PET	DC	^124^I	Monitor bone marrow-derived dendritic cell migration and antitumor effects	(105)
MRI	Neutrophils	SPIO	Provide a safe and effective way to monitor neutrophil dynamics	(115)
PET	Neutrophils	^18^F-CXCR2	Demonstrate specificity and diagnostic potential in neutrophil imaging	(116)
PET	MDSCs	^18^F-DPA-714	Characterize the heterogeneity of myeloid cell infiltration at different disease stages	(121)
MRI	MDSCs	^-^	Utilize a manganese dioxide coating and MDSC cell membrane camouflage to target the tumor microenvironment	(122)

### 3.2 Stromal cells

#### 3.2.1 MSCs

Mesenchymal stem cells (MSCs) are adult stem cells with self-renewal and multipotent differentiation capabilities, initially isolated from bone marrow but also present in adipose tissue, umbilical cord, placenta, and dental pulp (123). MSCs can differentiate into various cell types, including osteocytes, chondrocytes, and adipocytes, making them highly valuable in tissue engineering and regenerative medicine (124). Additionally, MSCs possess potent immunomodulatory functions, inhibiting immune cell activity and inflammatory responses, and have potential applications in transplant medicine, such as preventing graft-versus-host disease (GVHD). In the tumor microenvironment, MSCs play a particularly notable role. They exhibit a natural tumor-tropic property, enabling them to migrate to tumor sites, making them ideal carriers for targeted delivery of anticancer drugs, genes, and nanomaterials (125). For instance, MSCs can carry the sodium/iodide symporter (NIS) gene, driven by the IL-6 promoter, significantly enhancing glioblastoma (GBM) uptake of radioactive tracers, thereby improving imaging diagnostics and therapeutic outcomes. Studies have shown that mice treated with IL-6-NIS-MSCs and subsequent ^131^I therapy experienced significant tumor growth delay and increased survival rates (126). MSCs also play a crucial role in regulating immune responses within the tumor microenvironment. They secrete various cytokines and growth factors that modulate immune responses, suppressing the activity of T cells, B cells, NK cells, and macrophages, and reducing inflammation. This characteristic makes MSCs highly promising for tumor therapy. However, despite their therapeutic potential, some studies indicate that MSCs might promote tumor growth and metastasis under certain conditions. By secreting growth factors and matrix metalloproteinases (MMPs), MSCs can enhance tumor cell proliferation, angiogenesis, and invasion (127). This mechanism is complex and depends on the tumor type, MSC source, and specific tumor microenvironment.

Furthermore, the combination of MSCs with nanotechnology has shown significant promise. For example, a conductive nanocomposite hydrogel composed of gold nanorods and synthetic silicate nanoplatelets (GNR@SN/Gel) has been developed for myocardial infarction (MI) treatment. This hydrogel provides electromechanical coupling and allows PET/CT imaging to monitor implantation sites. The use of MSCs with this hydrogel has shown protective effects on myocardial survival and cardiac function, indicating great potential in MI therapy (128). Another study used ^89^Zr-indium oxine labeling and PET-CT imaging to demonstrate the distribution of MSCTRAIL cell therapy in a lung cancer mouse model. This method enables non-invasive tracking of MSCTRAIL without affecting the MSC phenotype and therapeutic efficacy, offering a potential approach for evaluating novel cell therapies, understanding their mechanisms, migration dynamics, and patient variability (129). MSCs play complex and multifaceted roles in the tumor microenvironment, acting as effective therapeutic tools but also potentially promoting tumor progression in certain scenarios.

#### 3.2.2 CAFs

In the tumor microenvironment, stromal cells, particularly cancer-associated fibroblasts (CAFs), promote tumor progression and therapeutic resistance by producing extracellular matrix (ECM) components and secreting cytokines and growth factors (130). Stromal cells also interact with immune cells, influencing their function and fostering an immunosuppressive environment. Molecular imaging techniques, such as PET and MRI, can monitor these cells’ activities in real-time, providing critical diagnostic information. Radiolabeled imaging combined with radioligands can accurately pinpoint tumor areas, supporting personalized treatment (131). For example, ^177^Lu-PSMA-617 radioligand therapy is a targeted treatment for prostate cancer patients expressing prostate-specific membrane antigen (PSMA). The use of radiolabeled imaging techniques such as ^177^Lu-PSMA-617 helps identify PSMA-positive tumors, enabling clinicians to administer targeted therapy based on the specific characteristics of the tumor (132). High-throughput spatial molecular imaging technologies, such as CosMx™, can detect thousands of RNA species at the single-cell level, offering detailed spatial analysis of the complex functions of stromal cells (133). Overall, molecular imaging significantly enhances the effectiveness of tumor diagnosis and treatment by precisely locating and monitoring stromal cells (134).

Cancer-associated fibroblasts (CAFs) are a significant and dynamic component of the tumor microenvironment (TME), playing crucial roles in tumor progression, metastasis, immune regulation, and therapeutic resistance. CAFs originate from various sources, including resident fibroblasts, epithelial-mesenchymal transition (EMT), and bone marrow-derived mesenchymal stem cells (MSCs) (135). CAFs support tumor cell proliferation and invasion by secreting growth factors, cytokines, and ECM components while remodeling the ECM to create barriers that facilitate tumor cell migration and protect them from therapeutic agents (136). They also create an immunosuppressive microenvironment by secreting immunosuppressive cytokines and chemokines, inhibiting the activity of cytotoxic T cells and NK cells, and promoting immune evasion (137). Additionally, CAFs enhance cancer stem cell survival and proliferation, induce metabolic reprogramming in tumor cells, and increase their resistance to chemotherapy and radiotherapy (138). The heterogeneity and plasticity of CAFs enable them to adapt to various signals in the TME, exhibiting different functional states. Targeting CAFs is a potential cancer therapy strategy aimed at reducing tumor growth, enhancing efficacy, and overcoming therapeutic resistance (139).

**Figure 3:**
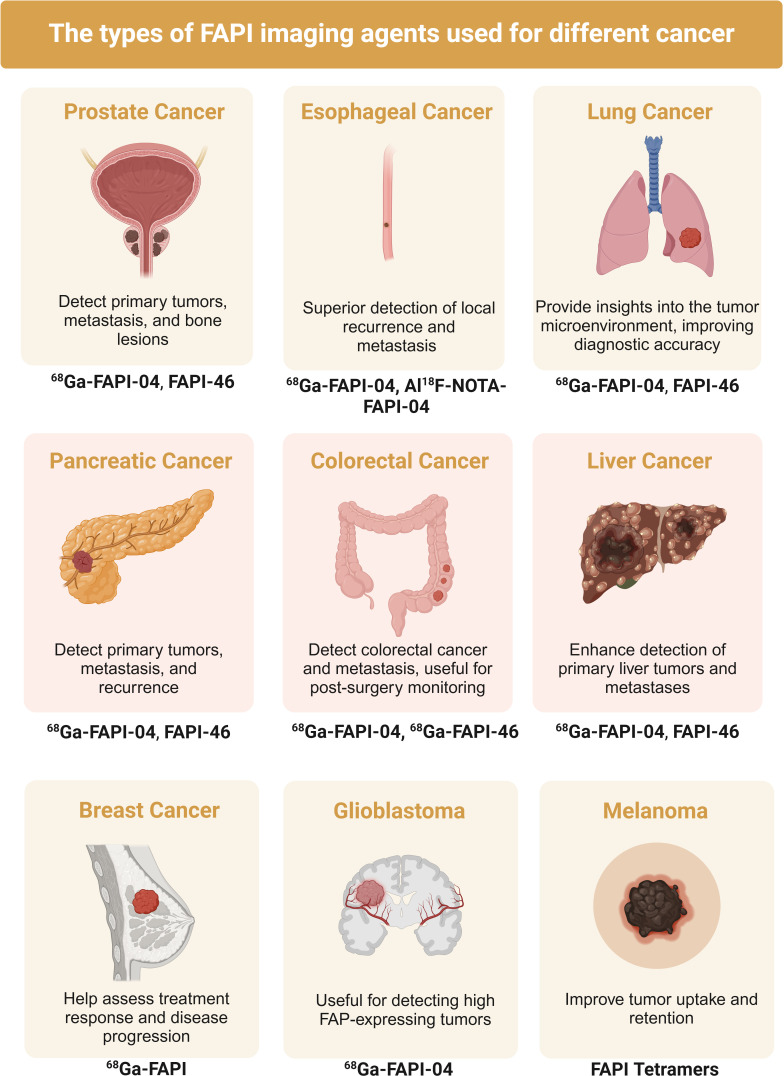
Published studies of fibroblast activating protein inhibitor positron emission tomography (FAPI) in the diagnosis of various types of cancer.

In recent years, imaging technologies targeting CAFs have made significant advances, greatly improving cancer diagnosis and treatment capabilities. One such technology involves [^99m^Tc] Tc-HYNIC-FAPI, a novel molecular probe specifically targeting CAFs by binding to fibroblast activation protein (FAP), demonstrating high specificity and sensitivity for SPECT/CT imaging (140). This technology excels in accurately locating and quantifying CAFs, aiding in understanding their role in tumor progression. IR-780 dye, a near-infrared dye, selectively accumulates in the mitochondria of cancer cells and CAFs, enhancing cancer immunotherapy by increasing T lymphocyte infiltration and serving imaging purposes (141). FAP-targeting ligands like PNT6555 deliver therapeutic agents directly to the tumor stroma by binding to FAP, increasing drug concentration and reducing side effects, offering both imaging and therapeutic functions (142). FAP-targeting radioligands such as FAP8-IP-DOTA exhibit high affinity and specificity, providing high-contrast tumor microenvironment images and prolonged retention in tumors, suitable for precise tumor localization and repeated treatment (143). Albumin-binding FAP radioligands like ^68^Ga-FSDD3I further enhance tumor uptake and retention time, offering high-quality imaging in PET scans (144). Researchers have studied the use of ^68^Ga-FAPI PET/CT imaging to monitor the effectiveness of combining a TGF-β receptor (TGF-βR) inhibitor with immunotherapy for treating metastatic colorectal cancer (CRC). The study showed that ^68^Ga-FAPI PET/CT imaging can accurately monitor the dynamic changes in CAFs and tumor immune responses. The TGF-βR inhibitor enhanced tumor-infiltrating T cells, increasing CRC sensitivity to KN046, a drug that blocks both PD-L1 and CTLA-4. This study supports the use of dual ^68^Ga-FAPI and ^18^F-FDG PET/CT imaging in managing CRC (145).These advancements provide new hope for early cancer detection, precise localization, and personalized treatment, promising to significantly improve cancer diagnosis and treatment outcomes (**Figure 3**).

### 3.3 Markers of secreted factors and associated proteases targeting mesenchymal cells

#### 3.3.1 Integrins

Integrins are transmembrane receptors that facilitate cell adhesion to the ECM and play a crucial role in cell migration, invasion, survival, and immune responses. Their role in tumor immunity has been extensively studied, revealing their dual functions in promoting and inhibiting immune responses within TME (146). Integrins are essential for the migration and infiltration of immune cells. For example, integrin αLβ2 (LFA-1) on T cells interacts with ICAM-1 on endothelial cells, promoting T cell migration to tumors (147). Integrins also play a key role in the activation and function of immune cells. For instance, integrin αMβ2 (Mac-1) on macrophages mediates phagocytosis and antigen presentation. Furthermore, integrins are vital in recruiting and polarizing TAMs, which can either support or inhibit tumor growth. Integrins can also interact with immune checkpoint molecules, influencing their expression and function (148). Blocking integrin interactions can reduce PD-1 and CTLA-4 expression, thereby enhancing T cell activation and anti-tumor responses (149). Inhibitors or antibodies targeting integrins have emerged as promising strategies in cancer immunotherapy.

Integrins are transmembrane receptors that promote cell adhesion to the ECM, playing a critical role in tumor growth and metastasis. Researchers have developed various integrin-targeting imaging probes, validating their efficacy in preclinical and clinical studies. One such probe is Ga-68-Trivehexin, developed to target integrin αvβ6. This probe has shown high affinity and selectivity across multiple cancers, demonstrating high-specific uptake in metastatic PDAC and head and neck squamous cell carcinoma (HNSCC) in clinical PET/CT imaging. These findings suggest that Ga-68-Trivehexin holds great potential for imaging tumors expressing integrin αvβ6 (150). Another study developed a heterodimeric peptide targeting vascular endothelial growth factor receptor (VEGFR) and integrin αv, labeled with zirconium-89 (^89^Zr) for PET imaging of gliomas. This probe exhibited strong binding capability and clear tumor visualization in a mouse glioma xenograft model. Its dual-targeting characteristic provided superior imaging performance over single-targeting probes, showcasing the advantages of multi-target probes in tumor imaging (151). Additionally, the clinical evaluation of ^68^Ga-FAPI-RGD, a PET probe simultaneously targeting fibroblast activation protein (FAP) and integrin αvβ3, has demonstrated higher tumor uptake and tumor-to-background ratio (TBR) compared to traditional ^18^F-FDG PET/CT. This probe offers better lesion detection and tumor delineation, particularly in diagnosing lymph node and bone metastases, highlighting its potential as a new tool for cancer imaging (152). An exploratory study also assessed the effectiveness of 68Ga-FAPI-RGD in PET/CT imaging of patients with suspected malignant lung tumors. The results showed that ^68^Ga-FAPI-RGD had a higher primary lesion detection rate, increased tumor uptake, and better accuracy in evaluating mediastinal lymph nodes compared to ^18^F-FDG and single-target probes ^68^Ga-RGD and ^68^Ga-FAPI. This indicates that dual-targeting probes significantly improve lung cancer diagnosis and staging (153). These advancements highlight the critical role of integrins in cancer molecular imaging, providing new perspectives for future diagnostics and treatments. By developing novel imaging probes targeting integrins, scientists can more accurately detect and monitor tumors, optimize treatment plans, and ultimately improve patient outcomes.

#### 3.3.2 MMPs

Matrix metalloproteinases (MMPs) play a crucial role in tumor immunity by regulating ECM degradation, cell migration, and signal transduction within TME. MMPs degrade ECM components, promoting tumor cell invasion and metastasis, and release growth factors and cytokines stored in the ECM, altering the immune environment of the TME (154). MMP-2 and MMP-9 degrade type IV collagen, increasing the availability of angiogenic factors, and promoting tumor angiogenesis, which provides nutrients and oxygen to tumor cells. Additionally, MMPs affect the function and migration of immune cells (155). MMP-9 has been shown to regulate T cell migration and activation, influencing their infiltration and anti-tumor activity. TAMs also express high levels of MMPs, promoting the formation of an immunosuppressive TME and inhibiting anti-tumor immune responses (156). Targeted inhibition of MMPs can reactivate the anti-tumor functions of immune cells and enhance the effectiveness of immunotherapy.

Researchers have developed a dual-modality (PET/NIRF) imaging agent targeting MT1-MMP, which is overexpressed in high-grade sarcomas. This imaging agent, [^89^Zr] Zr-DFO-anti-MT1-MMP-IRDye800CW, demonstrates high specificity for MT1-MMP, allowing for preoperative planning and intraoperative guidance, significantly improving surgical outcomes in a sarcoma mouse model. This indicates its important application value in enhancing the success rate of sarcoma surgeries (157). Another study explored a fluorinated derivative of mangiferin (MF) as a potential PET radiopharmaceutical. They found that F-propyl-MF can form a stable complex with the catalytic zinc atom of MMP-9, demonstrating its potential as a PET imaging agent. This research provides a theoretical basis for the development of MMP-9-targeted PET imaging agents (158). Furthermore, a dual-modality imaging probe targeting MMP-14, overexpressed in glioma cells, was developed. This probe combines a PET radionuclide with near-infrared fluorescence (NIRF) dye, activating the fluorescent signal upon MMP-14 cleavage, improving the visualization and differentiation of glioma cells. In preclinical models, this probe significantly enhanced tumor imaging, offering a new tool for non-invasive imaging and intraoperative navigation (159). Additionally, a multifunctional PET/MRI nanoparticle was designed to image MMP-2 activity in atherosclerotic plaques. The nanoparticle, ^64^Cu-NOTA-IONP@MMP2c-PEG2K, showed high uptake and excellent imaging contrast in macrophage-rich plaques. This smart nanoparticle can non-invasively assess MMP-2 activity *in vivo*, providing detailed molecular imaging information through combined PET and MRI, aiding in early diagnosis and therapeutic intervention (160). By developing novel imaging probes targeting MMPs, scientists can more accurately detect and monitor tumors, optimize treatment plans, and ultimately improve patient outcomes.

### 3.4 Cytokines

Cytokines are small signaling proteins that play a crucial role in tumor development by regulating immune responses and cell-to-cell communication. Cytokines secreted by NK cells, such as IFN-γ and TNF-α, can enhance their anticancer functions (161). TAMs in ovarian cancer secrete cytokines like IL-6 and TGF-β, which promote tumor invasion and metastasis (162). In prostate cancer, the androgen receptor signaling pathway regulates cytokines, affecting immune-mediated antitumor responses (163). Neutrophil functions within the tumor microenvironment are influenced by cytokines, displaying both pro-tumor and anti-tumor roles (164). Radiolabeled anti-PD-L1 monoclonal antibodies used in PET imaging can non-invasively assess PD-L1 expression levels, predicting the efficacy of immunotherapy (165). In patients with schistosomiasis-related bladder cancer, levels of pro-inflammatory cytokines such as IL-1β, IL-6, and TNF-α are elevated (166). Cytokines also play a significant role in the interactions between cancer stem cells and the immune system (167). These studies demonstrate that cytokines secreted by immune cells are vital in modulating the tumor microenvironment, significantly impacting tumor growth, invasion, and therapeutic response.

In recent years, significant progress has been made in understanding the role of cytokines secreted by immune cells in the tumor microenvironment and their application in PET imaging. These studies have not only enhanced our understanding of cytokine functions but also provided new tools for precise cancer diagnosis and treatment. PD-L1 PET imaging has been used to assess PD-L1 expression in tumors, a key immunosuppressive cytokine secreted by tumor cells and tumor-infiltrating immune cells. By using radiolabeled anti-PD-L1 monoclonal antibodies (e.g., ^89^Zr-atezolizumab), researchers can non-invasively image PD-L1 expression levels and evaluate their relationship with immunotherapy response (168). Additionally, IL-2, another crucial cytokine primarily secreted by activated T cells, promotes the proliferation and activation of T cells and NK cells. Radiolabeled IL-2 (e.g., ^18^F-IL-2) has been used in PET imaging to monitor the activation state and distribution of immune cells, playing a vital role in evaluating cancer patients’ immune responses and optimizing immunotherapy strategies (169).

CXCL8 (also known as IL-8), a chemokine secreted by various cells including tumor and immune cells, primarily attracts neutrophils to tumor sites. PET tracers labeled with CXCL8 or its receptor can image neutrophil infiltration in tumors, which is crucial for studying tumor inflammation and the immunosuppressive microenvironment (170). CXCR3 is a chemokine receptor primarily expressed on activated T cells and natural killer cells, playing a role in inflammation and immune responses. A novel F-18-labeled small molecule PET tracer for CXCR3 imaging successfully detected CXCR3 expression in atherosclerotic mouse models (171). CXCR4, a chemokine receptor highly expressed in various tumor cells, is closely associated with tumor invasion and metastasis. In this study, researchers used a radiolabeled CXCR4 ligand for PET/CT imaging of nasopharyngeal carcinoma patients. The results showed that Ga-68-labeled CXCR4 PET/CT successfully localized and quantified CXCR4 expression levels in tumors, providing crucial information about tumor biology (172). CCR2 is critical for monocyte and macrophage migration. An 18F-labeled radiotracer targeting CCR2, used in PET imaging, revealed CCR2’s essential role in monocyte recruitment and inflammatory responses (173). CCR5, expressed on various immune cells including T cells, macrophages, and dendritic cells, is involved in HIV infection, inflammatory diseases, and cancer. Successful CCR5 PET imaging with ^64^Cu-DOTA-DAPTA-targeted nanoparticles effectively localized and quantified CCR5 expression (174). CMKLR1 regulates the activity of natural killer cells and dendritic cells under the mediation of the chemokine Chemerin. CMKLR1’s role in metabolic diseases, inflammation, and cancer makes it an emerging target for PET imaging. A PET tracer targeting CMKLR1 has been used to detect CMKLR1 expression in acute lung injury (175).The advancements in PET imaging technology for studying cytokines secreted by tumor immune cells have significantly deepened our understanding of the tumor microenvironment and opened new avenues for precise cancer treatment. Future research directions include developing more specific and sensitive PET tracers and combining PET with other imaging techniques for multimodal imaging.

### 3.5 Imaging for simultaneous targeting of different regions of TME

In recent years, significant advancements in imaging technologies have enabled the simultaneous targeting of multiple regions within TME. These developments are crucial for understanding the complex interactions among tumor cells, the immune system, stromal cells, and blood vessels, all of which contribute to cancer progression and therapeutic resistance. The integration of different imaging modalities allows for more comprehensive and precise analysis of the TME, enhancing the ability to monitor tumor dynamics in real-time and guide targeted therapies effectively.

Researchers have developed hybrid imaging techniques by combining modalities such as CT, MRI, FLT. These multimodal imaging systems enable the simultaneous visualization of multiple components of the TME, including tumor vasculature, immune cells, and stromal elements. For example, combining CT and MRI with FLT improves biodistribution analysis and tumor characterization, offering superior soft tissue contrast that makes it easier to identify tumor boundaries and track therapeutic agents more effectively (176). Additionally, the integration of functional imaging modalities allows for the identification of tumor-associated changes, such as alterations in perfusion or metabolic activity. By targeting multiple regions of the TME simultaneously, these imaging techniques provide a more comprehensive view of the tumor’s biological environment.

Another significant advancement is the development of multiplexed imaging technologies, such as Imaging Mass Cytometry (IMC). This technique allows for the simultaneous assessment of multiple markers at the single-cell level, providing a deeper understanding of the cellular composition and spatial organization of the TME. Researchers have utilized IMC to analyze a variety of markers within the TME, revealing insights into the interactions between immune and stromal components (177). By characterizing the immune context and stromal features of the tumor, IMC offers strong evidence for studying tumor heterogeneity, which is critical for understanding treatment responses and identifying new therapeutic targets.

By simultaneously targeting and visualizing multiple components of the TME, clinicians can gain a more comprehensive understanding of tumor behavior, which aids in developing more personalized and effective treatment strategies.

## 4 Challenge and future

Molecular imaging technologies are increasingly important in cancer diagnosis, treatment, and monitoring, especially in the context of immunotherapy. However, these technologies face several challenges that need to be addressed to enhance their clinical utility. Additionally, the future prospects of molecular imaging in tumor immunotherapy offer promising opportunities to improve patient outcomes. One of the main challenges of molecular imaging is the complexity and sensitivity of these technologies. Techniques such as PET, MRI, and SERS (surface-enhanced Raman spectroscopy) require high sensitivity and specificity to detect molecular changes at the cellular level. The complexity of tumor biology, including the heterogeneity of cancer cells and the dynamic nature of the tumor microenvironment, makes it difficult to develop imaging agents that can precisely target specific biomarkers without generating non-specific background signals. For instance, while SERS-based biosensors show promise, their application in the early diagnosis of cancers like pancreatic cancer still faces significant obstacles (178). Another notable challenge is the limited availability of multimodal imaging agents. These agents combine the advantages of various imaging modalities to provide comprehensive insights, such as integrating the high spatial resolution of MRI with the functional imaging capabilities of PET. However, creating stable and biocompatible imaging agents that work effectively across multiple modalities is a complex process. Recent research on pH-targeted molecular imaging techniques, such as pHLIP-anchored imaging agents, has shown potential in tumor diagnosis and therapy, but there remains a need for more versatile and efficient imaging agents in clinical settings (179, 180). Additionally, the integration and interpretation of the vast amounts of data generated by molecular imaging technologies present a significant challenge. The exponential growth of data requires robust, standardized frameworks for analysis and interpretation. Artificial intelligence (AI) and machine learning (ML) have been proposed as solutions, particularly for analyzing complex multimodal datasets. However, the clinical integration of AI tools, such as graph neural networks (GNNs), is still in its early stages (181, 182).

The future of molecular imaging is closely linked to advancements in AI and ML. These technologies are expected to play a critical role in improving image analysis and interpretation, helping to overcome challenges such as image noise, variability in imaging protocols, and the subjectivity of image interpretation. By training AI models on large datasets, predictive algorithms can be developed to assist clinicians in making more accurate diagnostic and treatment decisions. For example, deep learning technologies are being explored for the early detection of lung cancer and lung nodules, aiming to improve diagnostic accuracy and efficiency (183). Another promising research area is the development of novel imaging agents that target specific molecular pathways and provide real-time insights into tumor biology. These imaging agents must be highly specific, stable, and capable of delivering clear contrast in imaging studies. Activatable multimodal probes that respond to specific physiological changes in the tumor microenvironment, such as pH shifts or the presence of particular enzymes, are gaining attention for *in vivo* imaging and theranostics. These probes can be activated under specific conditions, providing more targeted and accurate imaging (184). The clinical translation and regulatory approval of molecular imaging technologies remain significant challenges. Bringing novel imaging agents and technologies from the lab to the clinic requires rigorous testing, including preclinical studies, clinical trials, and regulatory approvals. This process is often lengthy and costly, potentially delaying the availability of new imaging technologies for patients. Therefore, streamlining these processes is crucial for accelerating the clinical application of molecular imaging technologies. Molecular imaging plays a critical role in tumor immunotherapy, particularly in the management of immune checkpoint inhibitors (ICIs). PET/CT molecular imaging using [^18^F]-FDG has shown significant promise in evaluating the tumor microenvironment, detecting immune-related adverse events, assessing treatment efficacy, and predicting outcomes. This technology enables the non-invasive monitoring of immune responses and the identification of biomarkers that guide personalized immunotherapy strategies (185). Furthermore, novel molecular imaging tracers are making whole-body visualization through PET possible, allowing for the assessment of tumor and immune cell characteristics, which aids in making treatment decisions for tumor immunotherapy. These tracers provide insights into drug distribution and immune checkpoint molecules, which are crucial for optimizing treatment strategies and improving patient outcomes (186).

Advancements in imaging technologies have significantly enhanced the ability to monitor tumor responses to immunotherapy, facilitating more personalized treatment strategies. Among these, Molecular MRI has emerged as a key tool for non-invasively assessing tumor responses, detecting early signs of treatment failure, and providing insights into the biological effects of therapy. Additionally, this technique benefits from the integration of AI, which helps interpret complex imaging data and improves the accuracy of immunotherapy response assessments (187). Another promising approach is the use of non-immunogenic imaging techniques, such as murine sodium iodide symporter (mNIS) for SPECT imaging. This method provides a sensitive way to monitor tumor responses in immune-competent models, overcoming the immunogenicity issues commonly associated with traditional imaging techniques, thus enabling more accurate evaluations of immunotherapy efficacy (188). Furthermore, PET/CT imaging plays a crucial role in monitoring responses to immunotherapy, especially in breast cancer, where it helps assess tumor heterogeneity and track treatment efficacy. This imaging modality is essential for evaluating therapeutic responses and identifying patterns that can guide personalized treatment plans (28). Collectively, these advanced imaging technologies are critical for optimizing tumor immunotherapy, enhancing our ability to tailor treatments based on real-time, patient-specific tumor characteristics.

One of the major challenges in harnessing NK cells for cancer immunotherapy is their reduced numbers in the TME. Studies have shown that NK cells often undergo depletion in the TME, primarily due to immunosuppressive factors such as TGF-β, hypoxia, and suppressive immune cells like regulatory T cells (189). This poses a significant hurdle for effective NK cell-based therapies, as their reduced presence limits their ability to exert cytotoxic effects against tumor cells. For example, researchers have employed highly specific markers such as CD56 in combination with novel nanotechnology-based probes to enhance the detection of NK cells, even when their numbers are low. This approach significantly improves the sensitivity and precision of NK cell identification, making it feasible to study their dynamics in challenging conditions (190). In addition, strategies to expand NK cell populations *in vivo* are actively being explored to address the limitation of low cell numbers. These include cytokine-based therapies, such as IL-15 super-agonists, which promote NK cell proliferation and activation, as well as *ex vivo* expansion techniques prior to reinfusion, ensuring a robust population of functional NK cells for therapeutic applications (191, 192). Furthermore, advanced single-cell sequencing and high-throughput spatial molecular imaging technologies provide detailed insights into the functional states of NK cells within the ME. These methods not only facilitate the detection of NK cells but also enable a deeper understanding of their interactions within the TME (193). NK cells not only play a pivotal role in the treatment of solid tumors but also serve as a critical component in leukemia immunotherapy (189). For instance, the adoptive transfer of NK cells engineered to express the bone marrow homing receptor CXCR4^R334X^ has enhanced the targeting of leukemia cells residing in the bone marrow, thereby improving therapeutic efficacy (194). Similarly, CAR-NK cells designed to target specific leukemia antigens, such as CD19 and CD33, have demonstrated greater cytotoxicity and specificity against acute myeloid leukemia (AML) cells (195). Furthermore, advancements in molecular imaging technologies provide crucial support for NK cell-based therapies by enabling precise monitoring of NK cell migration, persistence, and interactions within the tumor microenvironment (196).

Molecular imaging has become an indispensable tool in cancer research and treatment, particularly in understanding and visualizing the complex interactions within the tumor microenvironment. Technologies that enable the non-invasive monitoring of immune cells, cytokines, and matrix components have brought significant advancements to the field, allowing for more precise and personalized treatment approaches. However, several challenges remain, including the development of more sensitive and specific imaging agents, the effective integration and interpretation of large datasets, and the translation of laboratory discoveries into clinical practice. The future of molecular imaging lies in overcoming these challenges through advances in artificial intelligence, the creation of innovative imaging probes, and the integration of these technologies with personalized medicine. Continued research and development in this field are essential for improving cancer diagnosis, monitoring treatment efficacy, and ultimately enhancing patient outcomes.
